# Distal cholangiocarcinoma mimicking pancreatic ductal adenocarcinoma successfully converted to resection following PD-1/CTLA-4 bispecific immunotherapy (cadonilimab) combined with nab-paclitaxel–based chemotherapy: a case report providing preliminary real-world insight

**DOI:** 10.3389/fonc.2026.1865799

**Published:** 2026-08-26

**Authors:** Yongzhao Li, Xiyang Sheng, Chen Mi, Yongyue Du, Siyang Wang, Dongdong Wang, Wei Wang, Longbo Wang, Gengyuan Shi, Mancai Wang, Tianlong Ma, Xuechao Xu, Yun Wang, Hanteng Yang

**Affiliations:** 1 Department of General Surgery, The Second Hospital of Lanzhou University, Academician Workstation of The Second Hospital & Clinical Medical School, Lanzhou University, Lanzhou, Gansu, China; 2 Department of Medical Oncology, Gansu Provincial Second People’s Hospital, Lanzhou, Gansu, China

**Keywords:** AG regimen, cadonilimab, conversion surgery, distal cholangiocarcinoma, dual immunotherapy, misdiagnosis, neoadjuvant therapy, pancreatic ductal adenocarcinoma

## Abstract

**Background:**

Distal cholangiocarcinoma (dCCA) and pancreatic ductal adenocarcinoma (PDAC) share highly overlapping clinical presentations and radiological features, often leading to preoperative misdiagnosis. Therapeutic strategies for these malignancies differ substantially. While PDAC is commonly treated with gemcitabine plus nab-paclitaxel (AG regimen) or FOLFIRINOX as neoadjuvant or conversion therapy, first-line treatment for cholangiocarcinoma (CCA) typically consists of gemcitabine plus cisplatin (GC regimen), with or without immunotherapy. In recent years, increasing attention has been paid to immunotherapy-based combination strategies in biliary tract cancers, although their role in conversion therapy remains to be fully elucidated.

**Case presentation:**

A 70-year-old male presented with upper abdominal discomfort. Imaging findings and endoscopic ultrasound-guided fine-needle aspiration (EUS-FNA) suggested moderately differentiated ductal adenocarcinoma. Based on the tumor location in the pancreatic uncinate process and associated vascular involvement, the lesion was initially considered borderline resectable PDAC. The patient received neoadjuvant therapy consisting of a programmed cell death protein 1 (PD-1) and cytotoxic T-lymphocyte-associated protein 4 (CTLA-4) bispecific antibody (cadonilimab) combined with the AG regimen for seven cycles. Post-treatment imaging demonstrated marked tumor regression and reduced vascular involvement. The patient subsequently underwent pancreaticoduodenectomy, with partial resection and reconstruction of the superior mesenteric vein (SMV), as well as arterial divestment of the superior mesenteric artery (SMA), achieving R0 resection. However, postoperative pathological evaluation revealed that the tumor originated from the distal bile duct, confirming a final diagnosis of dCCA.

**Conclusion:**

This case highlights the limitations of EUS-FNA in accurately determining the primary tumor origin in periampullary and pancreatobiliary junction malignancies, while immunohistochemistry lacks sufficient specificity in this setting. Importantly, it demonstrates that the AG regimen combined with PD-1/CTLA-4 bispecific immunotherapy, commonly used in PDAC, may exert significant antitumor activity in dCCA, providing a novel perspective for conversion therapy in biliary tract cancers. This report offers a preliminary real-world therapeutic insight from a single case, suggesting that dual immune checkpoint blockade combined with chemotherapy may enable conversion surgery in selected patients with CCA. Further studies are warranted to elucidate the underlying immunological mechanisms.

## 1 Introduction

Distal cholangiocarcinoma (dCCA) and pancreatic ductal adenocarcinoma (PDAC) are anatomically adjacent and share highly overlapping clinical presentations and radiological features, making preoperative differentiation particularly challenging, especially for lesions located in the pancreatic head or uncinate process (1, 2). Endoscopic ultrasound-guided fine-needle aspiration (EUS-FNA) is an important diagnostic tool for pancreatic masses; however, in tumors arising from the pancreatobiliary junction, its primary value lies in confirming malignancy rather than accurately determining the tumor origin (3–5).

Currently, PDAC and cholangiocarcinoma (CCA) differ substantially in systemic therapeutic strategies. For PDAC, gemcitabine plus nab-paclitaxel (AG regimen) or FOLFIRINOX has become a cornerstone of neoadjuvant or conversion therapy in patients with borderline resectable or locally advanced disease (6–9). In contrast, gemcitabine plus cisplatin (GC regimen) has long been established as the standard first-line treatment for CCA and has increasingly evolved into combination regimens incorporating immunotherapy (10–12). However, in tumors of the pancreatobiliary junction, diagnostic uncertainty may lead to misclassification of the primary site, thereby influencing treatment selection and clinical outcomes.

Notably, distal dCCA and PDAC share certain molecular characteristics and features of the tumor microenvironment, which may provide a biological rationale for exploring cross-tumor therapeutic strategies. In this context, chemotherapy regimens derived from pancreatic cancer treatment, such as nab-paclitaxel–based combinations, have been investigated in biliary tract cancers and have demonstrated potential antitumor activity in a subset of patients (13, 14). Meanwhile, immunotherapy, particularly immune checkpoint inhibitors (ICIs), has demonstrated substantial clinical benefits across a variety of solid tumors in recent years. Dual blockade of programmed cell death protein 1 (PD-1) and cytotoxic T-lymphocyte-associated protein 4 (CTLA-4) can enhance antitumor immune responses through complementary immunoregulatory pathways, showing potential synergistic effects. Although this strategy remains under active investigation in biliary tract cancers, emerging evidence suggests that it may confer clinical benefit in selected patient populations (15–18).

However, reports on the use of PD-1/CTLA-4 bispecific antibodies combined with the AG regimen in CCA, particularly those achieving successful conversion surgery, remain scarce. Herein, we report a case of dCCA initially misdiagnosed as PDAC, in which neoadjuvant treatment with a PD-1/CTLA-4 bispecific antibody (cadonilimab) combined with the AG regimen resulted in successful conversion to resection. This case suggests that, in the context of diagnostic uncertainty at the pancreatobiliary junction, immunotherapy-based cross-tumor treatment strategies may offer potential clinical value under specific circumstances, providing preliminary clinical evidence for this therapeutic approach.

## 2 Case presentation

**Figure 1:**
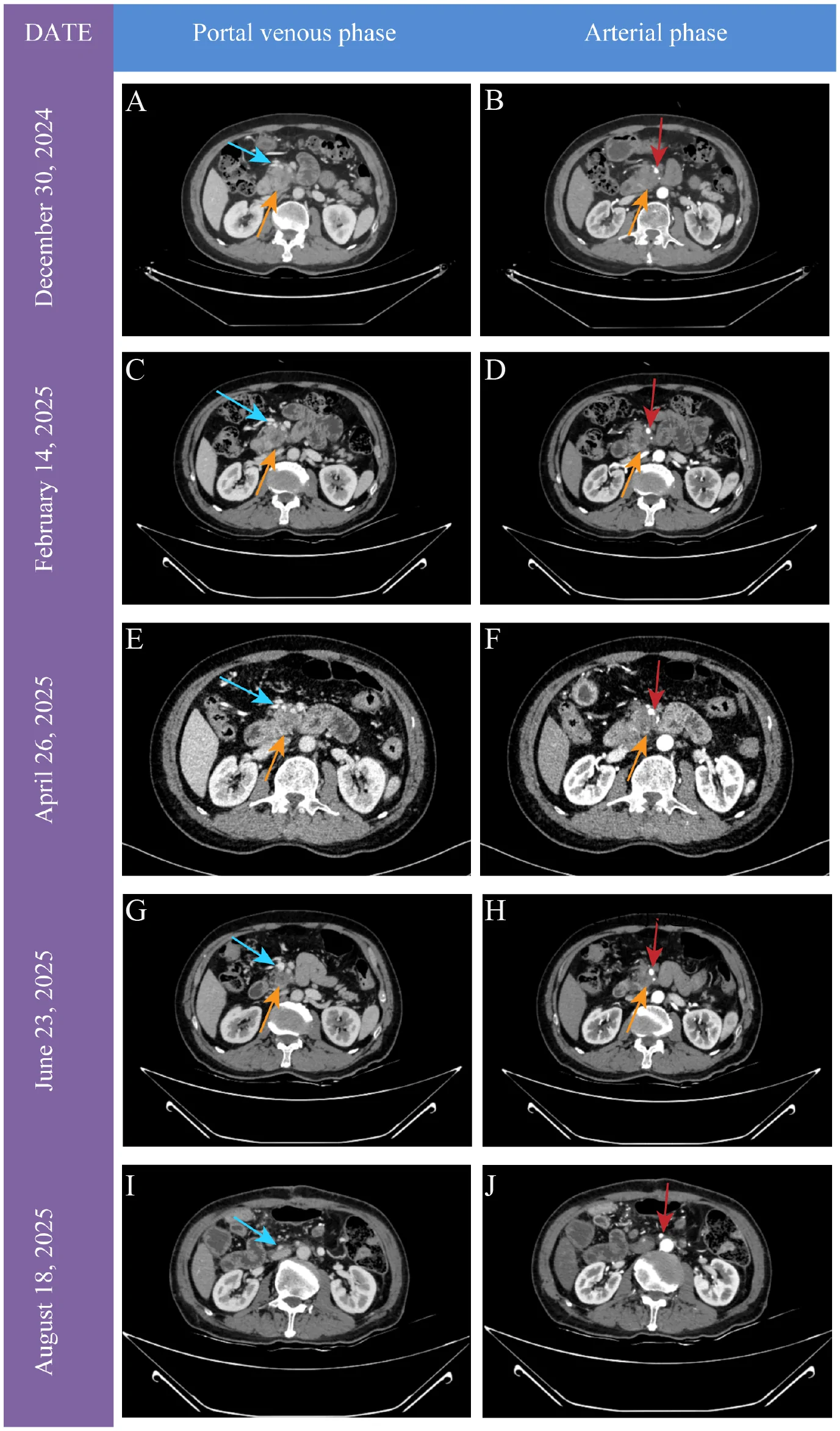
Serial contrast-enhanced CT images before and after surgery. **(A–H)** Four preoperative contrast-enhanced CT examinations (arterial and portal venous phases) demonstrated a marked reduction in the size of the primary tumor and alleviation of vascular involvement, including the SMV and SMA, following neoadjuvant therapy. The tumor is indicated by yellow arrows, SMV involvement by blue arrows, and SMA involvement by red arrows. **(I, J)** One postoperative follow-up contrast-enhanced CT scan showed no abnormal enhancement or enlarged lymph nodes after radical pancreaticoduodenectomy.

**Figure 2:**
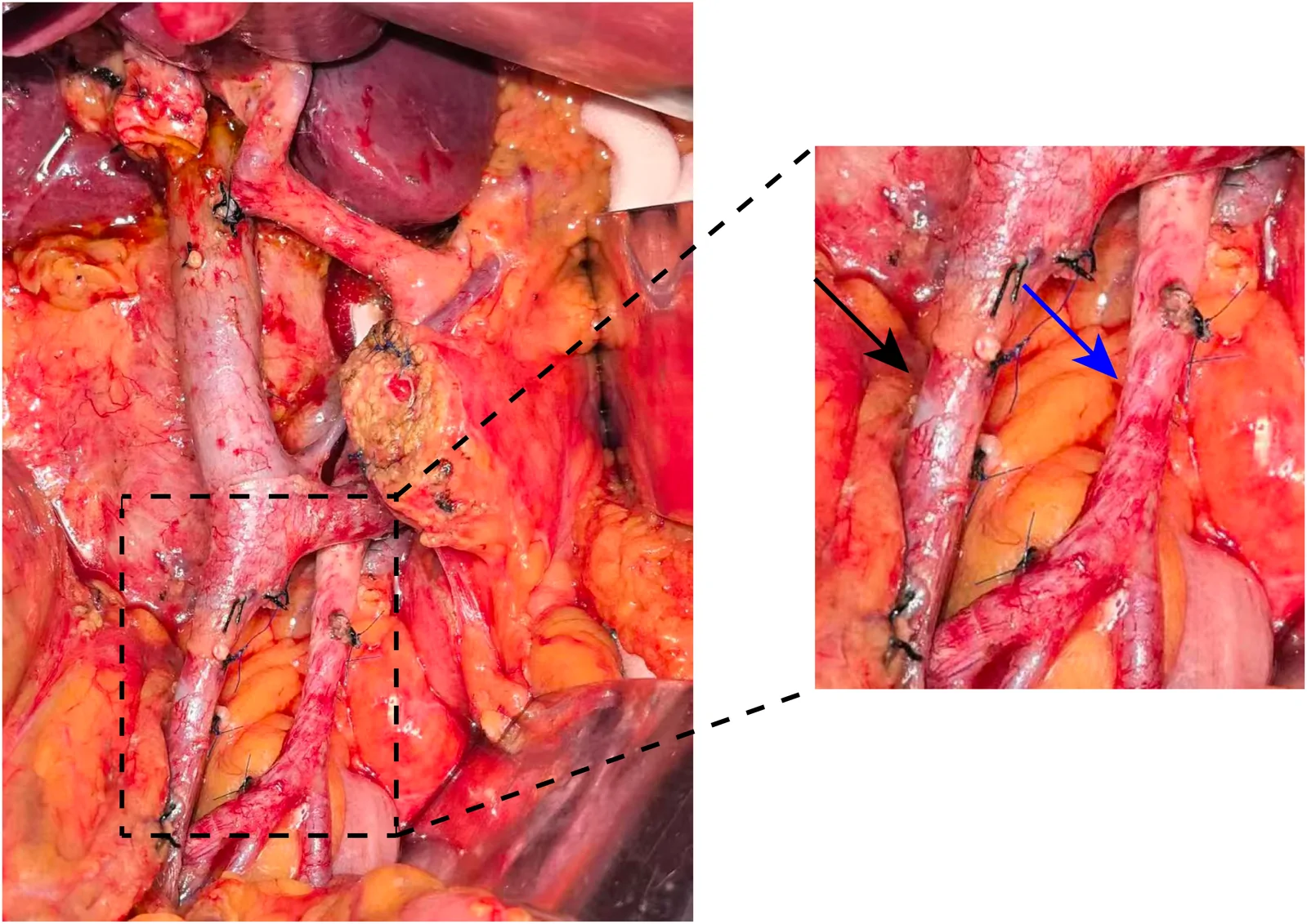
Intraoperative finding. The right image shows a magnified view of the left. The black arrow indicates the reconstructed SMV, and the blue arrow indicates the SMA following arterial divestment.

**Figure 3:**
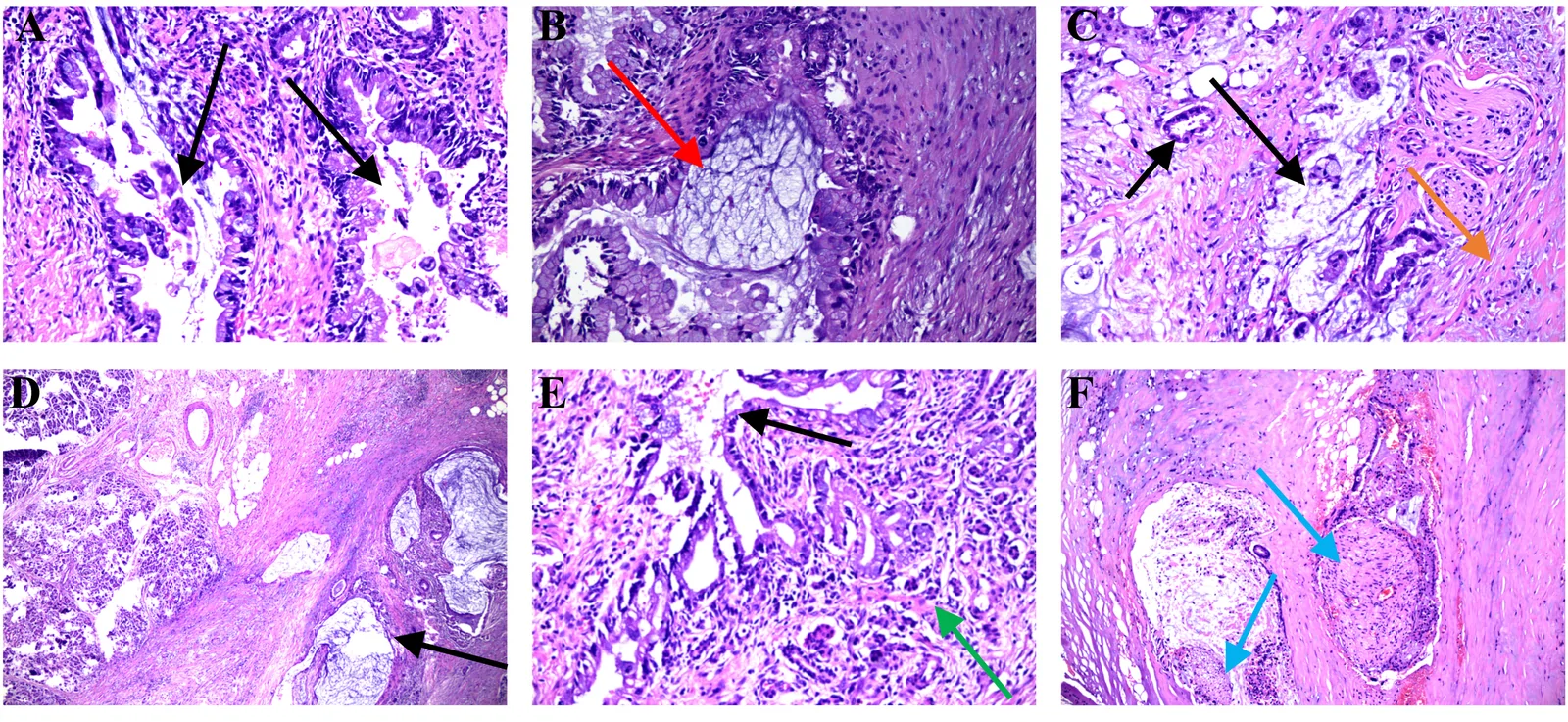
Histopathological examination of the resected specimen (hematoxylin and eosin staining). **(A–C)** Representative histopathological features of cholangiocarcinoma. The tumor is composed of infiltrative irregular glandular structures embedded within abundant desmoplastic stroma. Tumor cells consist of columnar and goblet cells with abundant eosinophilic or vacuolated cytoplasm, enlarged hyperchromatic nuclei, and marked cytological atypia. Focal mucinous components are present within the tumor (original magnification ×400). Black arrows indicate infiltrative tumor glands; red arrows indicate mucinous components; yellow arrows indicate desmoplastic stroma. **(D)** Low-power view demonstrating infiltrative growth of cholangiocarcinoma into the pancreatic parenchyma (×40). Black arrows indicate infiltrative tumor glands. **(E)** High-power view showing infiltrative tumor glands invading the pancreatic parenchyma (×400). Black arrows indicate infiltrative tumor glands; green arrows indicate pancreatic parenchyma. **(F)** Representative image showing tumor involvement of adjacent neurovascular structures, consistent with perineural and/or lymphovascular invasion (×100). Blue arrows indicate neurovascular structures involved by tumor cells.

A 70-year-old male was admitted with a 1-month history of persistent upper abdominal distension accompanied by intermittent abdominal pain. He denied jaundice, fever, nausea, vomiting, or significant weight loss. His past medical history was unremarkable. Physical examination revealed no obvious abnormalities. Magnetic resonance cholangiopancreatography (MRCP) performed at an outside hospital showed cholecystitis, cholelithiasis, bile duct dilation, and a suspicious lesion in the distal common bile duct, along with an abnormal signal in the pancreatic head. Contrast-enhanced computed tomography (CT) suggested a nodular lesion in the pancreatic uncinate process, raising suspicion for PDAC. Repeat enhanced CT at our institution demonstrated a 34 × 34 × 27 mm mass in the uncinate process with involvement of the superior mesenteric vein (SMV) and superior mesenteric artery (SMA), as well as enlarged retroperitoneal lymph nodes (**Figure 1**). Given that the lesion appeared to be centered within the pancreatic uncinate process and was associated with major peripancreatic vascular involvement, the imaging findings were considered highly suggestive of borderline resectable PDAC rather than a primary distal bile duct malignancy. Serum tumor marker evaluation revealed an elevated CA19–9 level of 101 U/mL, whereas CEA, CA125, and AFP levels were all within the normal range. To establish a definitive diagnosis, endoscopic ultrasound-guided fine-needle aspiration (EUS-FNA) was performed, revealing moderately differentiated ductal adenocarcinoma. Immunohistochemistry showed CK8/18 (+), CKpan (+), DPC4 (−), Syn (−), and P504S (−), with a Ki-67 index of approximately 30%. Based on these findings, the lesion was considered borderline resectable PDAC. Following multidisciplinary team (MDT) discussion, neoadjuvant therapy was recommended due to vascular involvement and the low likelihood of achieving R0 resection with upfront surgery. The patient received seven cycles of a PD-1/CTLA-4 bispecific antibody (cadonilimab) combined with the AG regimen (gemcitabine 1.3 g and nab-paclitaxel 170 mg). During treatment, serial monitoring demonstrated a gradual decline in serum CA19–9 levels, from 101 U/mL at baseline to 73 U/mL after the first cycle of neoadjuvant therapy and 55.3 U/mL after the fourth cycle. Prior to surgery, following completion of seven treatment cycles, the CA19–9 level had further decreased to 38.5 U/mL. All other tumor markers remained within normal limits throughout the treatment course. The treatment was well tolerated, with only mild fatigue and gastrointestinal discomfort, and no severe adverse events were observed. Post-treatment CT demonstrated significant tumor regression, with the lesion decreasing in size from 34 × 34 × 27 mm at baseline to approximately 25 × 23 mm after neoadjuvant therapy. Based on RECIST 1.1 criteria, the longest tumor diameter decreased from 34 mm to 25 mm, corresponding to a 26.5% reduction. In addition, vascular involvement was markedly reduced and regional lymph nodes decreased in size (**Figure 1**). Although the reduction in longest diameter did not reach the ≥30% threshold required for a RECIST-defined partial response, the overall radiographic findings suggested a favorable treatment response and conversion to resectable disease. Re-evaluation by the MDT suggested that conversion surgery was feasible. On June 29, 2025, The patient subsequently underwent pancreaticoduodenectomy. Intraoperatively, the tumor was located at the junction of the pancreatic head and distal bile duct and was adherent to the SMV, necessitating partial resection and reconstruction of the SMV (**Figure 2**). Although no definite arterial wall invasion of the SMA was observed, perivascular soft tissue involvement required arterial divestment (**Figure 2**). No distant metastases were identified, and R0 resection was successfully achieved. Postoperative pathology revealed a moderately differentiated adenocarcinoma of the common bile duct with focal mucinous features, measuring approximately 3.5 × 3 × 2 cm. The tumor infiltrated the full thickness of the bile duct wall with secondary invasion into the pancreas, without duodenal involvement. Perineural and lymphovascular invasion were identified, and all resection margins were negative (**Figure 3**). Lymph node examination demonstrated metastasis in 1 of 13 pericholedochal lymph nodes, with no metastasis detected in other nodal groups. According to the 8th edition of the AJCC staging system, the tumor was staged as pT3N1M0 (19). 5Immunohistochemical analysis performed on the resected specimen as part of routine pathological evaluation showed: CK7 (+), focal CK20 (+), CDX-2 (−), strong TROP2 expression, loss of DPC4 expression, MUC5AC (+), scattered MUC6 (+), MUC2 (−), and S100P (+). p53 showed a wild-type pattern. The Ki-67 index was approximately 45%, and PD-L1 expression yielded a combined positive score (CPS) of 30. Based on the tumor location in the distal common bile duct and its morphological features, the final diagnosis was dCCA, rather than the preoperative diagnosis of PDAC. The patient had an uneventful postoperative recovery and was discharged on postoperative day 13. Given the pathological findings of pT3N1M0 disease with perineural and lymphovascular invasion, postoperative adjuvant therapy was discussed by the MDT. However, because an R0 resection had been achieved and considering the patient’s advanced age (70 years), satisfactory postoperative recovery, potential treatment-related toxicity, and personal preference, no adjuvant chemotherapy, immunotherapy, or radiotherapy was administered. The patient was subsequently followed with regular surveillance. At follow-up in August 2025 at our institution, the patient was in good general condition, with no evidence of recurrence or metastasis (**Figure 1**), Serum CA19–9 had further decreased to 11.20 U/mL, which was within the normal range, and all other tumor markers remained unremarkable. At the most recent follow-up on May 21, 2026, 10 months and 22 days after surgery, the patient remained alive and in good general condition, with no reported evidence of tumor recurrence, distant metastasis, or late treatment-related adverse events. The recurrence-free survival time was 10.7 months. Since August 2025, follow-up examinations have been performed at a local hospital, and the original imaging data were not available for centralized review.

## 3 Discussion

DCCA and PDAC are anatomically adjacent and share substantial overlap in clinical presentation, radiological findings, and even certain pathological features. Consequently, preoperative differentiation between these two entities has long remained a major challenge in hepatobiliary surgery and gastrointestinal oncology. This difficulty is particularly pronounced when tumors are located in the pancreatic head or uncinate process and are accompanied by biliary dilation, peripancreatic infiltration, and involvement of adjacent major vessels, under which circumstances dCCA may closely mimic PDAC on imaging (1, 2, 20). The present case represents a typical example of this scenario: preoperative imaging demonstrated a lesion in the pancreatic uncinate process with involvement of the SMA and SMV, while biopsy findings supported ductal adenocarcinoma, leading to an initial clinical diagnosis of PDAC. However, postoperative examination of the resected specimen revealed that the tumor was centered in the distal common bile duct, with full-thickness invasion of the bile duct wall and secondary extension into the pancreatic parenchyma, thereby confirming its origin as dCCA. This “diagnostic reversal” highlights that, in tumors arising from the periampullary and pancreatobiliary junction regions, determination of the primary site should not rely solely on local imaging findings or limited biopsy results, but rather requires a comprehensive evaluation incorporating overall histopathological architecture and anatomical relationships.

The misdiagnosis in this case can be primarily attributed to the complexity of the local anatomical structures. DCCA typically exhibits an infiltrative growth pattern along the bile duct wall and may extend into adjacent fibrous tissue, neural plexuses, and pancreatic parenchyma. When the lesion arises in the distal common bile duct adjacent to the pancreatic head and uncinate process, imaging findings are more likely to resemble a pancreatic mass rather than the typical features of bile duct wall thickening or intraductal lesions. Furthermore, local inflammatory changes, fibrosis, and the intrinsic anatomical overlap at the pancreatobiliary junction further increase the difficulty of radiological interpretation. Therefore, even when contrast-enhanced CT or MRCP suggests that the lesion is primarily located in the pancreatic head or uncinate process, the possibility of dCCA with secondary pancreatic invasion should be carefully considered to avoid misinterpretation of imaging findings (1, 21). Apart from imaging findings, although EUS-FNA is an important pathological diagnostic tool for pancreatic mass lesions, its value in lesions arising at the pancreatobiliary junction lies more in confirming malignancy than in accurately identifying the primary site (4, 22, 23). In the present case, preoperative EUS-FNA suggested a moderately differentiated ductal adenocarcinoma. Immunohistochemistry showed CK8/18 positivity, CKpan positivity, wild-type p53 expression, loss of DPC4, negative Syn and P504S staining, and a Ki-67 index of approximately 30%. From a pathological perspective, these findings were sufficient to support the diagnosis of an epithelial-derived malignant ductal adenocarcinoma, but were insufficient to reliably distinguish PDAC from dCCA. The underlying reason is that PDAC and dCCA both belong to the pancreatobiliary adenocarcinoma spectrum and show substantial overlap in immunophenotypic features, often exhibiting the so-called pancreatobiliary phenotype (2, 24). Specifically, positivity for CKpan and CK8/18 primarily supports ductal epithelial differentiation; however, both markers are commonly expressed in PDAC and dCCA and lack sufficient site specificity. Similarly, wild-type p53 expression does not provide discriminatory value between these two entities (2, 25). Loss of DPC4 (SMAD4) is more frequently regarded as a feature suggestive of PDAC; nevertheless, it is not specific to PDAC and can also be observed in CCA. Therefore, in limited biopsy specimens, it is insufficient to determine the site of origin when used in isolation (26–29). On the other hand, negative Syn staining mainly helps exclude overt neuroendocrine differentiation, while P504S is not a core marker for differentiating PDAC from dCCA. Accordingly, both markers provide limited value in determining tumor origin in this context (30–32). Taken together, the principal diagnostic implication of the preoperative biopsy in this case was the confirmation of a malignant ductal adenocarcinoma, rather than definitive identification of a pancreatic origin. Postoperative immunohistochemical analysis of the resected specimen further highlighted this diagnostic challenge. The tumor demonstrated positivity for CK7, focal CK20, TROP2 (strong), MUC5AC, scattered MUC6, and S100P, with negativity for CDX-2 and MUC2. DPC4 expression was lost, p53 remained wild-type, the Ki-67 index was approximately 45%, and the PD-L1 CPS was 30. Overall, this immunoprofile is more consistent with a pancreatobiliary-type adenocarcinoma rather than a pattern highly specific to a single organ of origin (2, 20). CK7 positivity is commonly observed in both PDAC and dCCA, reflecting pancreatobiliary ductal differentiation but lacking site specificity. Focal CK20 expression is relatively more frequent in dCCA; however, its diagnostic contribution remains limited and is insufficient as a reliable discriminator between the two entities (25, 33, 34). Negative CDX2 and MUC2 expression generally argue against intestinal-type differentiation, thereby helping exclude intestinal-type ampullary or gastrointestinal-type periampullary adenocarcinomas. However, these markers provide limited value in distinguishing PDAC from dCCA (2, 33, 35). In contrast, positivity for MUC5AC and scattered expression of MUC6 suggest mucinous differentiation and a pancreatobiliary epithelial phenotype. While these features may lend some support to dCCA, they are also frequently observed in PDAC and therefore cannot be used to determine the site of origin (33). S100P positivity is a well-recognized marker associated with the pancreatobiliary lineage, indicating that the tumor belongs to this epithelial spectrum, but it similarly lacks sufficient organ specificity (26, 36, 37). High TROP2 expression is relatively common in biliary tract malignancies and primarily reflects epithelial malignancy and potential therapeutic relevance, rather than serving as a classical marker for site determination (38). In summary, this immunophenotypic profile is best interpreted as supporting a pancreatobiliary ductal adenocarcinoma lineage and, to some extent, excluding intestinal-type differentiation. However, it remains insufficient to definitively distinguish between PDAC and dCCA based solely on immunohistochemistry. Therefore, this case suggests that, for adenocarcinomas arising at the pancreatobiliary junction, conventional immunohistochemistry based on limited biopsy specimens is primarily useful for confirming ductal epithelial origin, identifying pancreatobiliary differentiation, and excluding special tumor subtypes. In contrast, determination of the primary site still requires comprehensive evaluation incorporating overall histological architecture, the anatomical center of the tumor, patterns of invasion, and imaging findings. In the present case, the final diagnosis of dCCA was not established based on any single immunohistochemical marker, but rather on the integrated assessment of the resected specimen. The tumor was centered in the distal common bile duct, with full-thickness involvement of the bile duct wall and secondary extension into the pancreas. Importantly, no morphological evidence supported a primary origin from the pancreatic ductal system with subsequent invasion into the bile duct. This case highlights that, in tumors arising at the pancreatobiliary junction, the determination of the primary site relies predominantly on overall histopathological features and anatomical localization, while immunohistochemistry plays a supportive role in confirmation and differential exclusion rather than serving as a definitive determinant. From a practical diagnostic perspective, this case also provides several lessons that may help reduce similar diagnostic pitfalls in future clinical practice. First, when a lesion is located at the pancreatobiliary junction, dCCA should remain an important differential diagnosis even when imaging suggests a mass centered in the pancreatic head or uncinate process. Second, imaging findings should be interpreted in conjunction with MRCP features, patterns of biliary involvement, and the anatomical relationship between the lesion and the distal bile duct, rather than relying solely on the apparent location of the mass. Third, clinicians should recognize that EUS-FNA and limited biopsy specimens are highly valuable for confirming malignancy but may have limited ability to determine the primary site of origin in this anatomical region. Therefore, multidisciplinary assessment integrating radiological, endoscopic, pathological, and anatomical information remains essential for improving diagnostic accuracy and reducing the risk of misclassification in tumors arising at the pancreatobiliary junction.

Beyond the diagnostic implications, this case is particularly informative from a therapeutic perspective. Currently, first-line treatment strategies differ substantially between PDAC and CCA. For PDAC, AG regimens or FOLFIRINOX are commonly used in the neoadjuvant or conversion setting (6–9), whereas for CCA, GC remains the standard first-line therapy (10–12), often in combination with immunotherapy. In this case, the lesion was initially diagnosed as borderline resectable PDAC based on imaging findings and EUS-FNA pathology. Accordingly, treatment decisions were made according to contemporary PDAC management principles, and the patient received AG chemotherapy combined with PD-1/CTLA-4 bispecific immunotherapy. Notably, this treatment approach resulted in a marked therapeutic response, including significant tumor shrinkage, reduced vascular involvement, and ultimately successful R0 resection. The decision to administer cadonilimab was based on the initial diagnosis of borderline resectable PDAC and the growing body of clinical evidence supporting dual immune checkpoint blockade in pancreatic cancer. Although immune checkpoint inhibitor monotherapy has generally shown limited efficacy in PDAC, simultaneous inhibition of the PD-1 and CTLA-4 pathways may enhance antitumor immune responses and partially overcome the highly immunosuppressive tumor microenvironment characteristic of PDAC. Furthermore, chemotherapy can promote tumor antigen release and immune cell recruitment, thereby generating synergistic antitumor effects when combined with immunotherapy. Cadonilimab is a PD-1/CTLA-4 bispecific antibody that enables dual checkpoint blockade while potentially reducing the incidence of immune-related adverse events. A recent study by Qin et al. demonstrated promising antitumor activity and manageable safety of cadonilimab combined with AG chemotherapy in patients with advanced PDAC, achieving a disease control rate of 85.7% and a median overall survival of 11.45 months (39). Taken together, under the initial clinical diagnosis of borderline resectable PDAC, the use of cadonilimab combined with AG chemotherapy was biologically rational and supported by emerging clinical evidence. Interestingly, despite the final pathological diagnosis of dCCA, this treatment strategy achieved a favorable response and enabled curative-intent resection in the present case. Previous studies have explored treatment strategies derived from PDAC regimens, such as nab-paclitaxel–containing chemotherapy, in biliary tract cancers, with some patients demonstrating antitumor activity. However, the overall level of evidence remains limited, and such approaches have not been established as standard treatment options (13, 14). The findings from the present case are, to some extent, consistent with these prior observations and further suggest that cross-tumor therapeutic strategies may have potential value in selected clinical contexts. Although direct evidence specifically addressing cross-tumor therapeutic strategies in periampullary malignancies remains scarce, similar treatment crossover has already been explored within the broader pancreatobiliary cancer spectrum. The activity observed with nab-paclitaxel–containing regimens in biliary tract cancers suggests that therapeutic approaches originally developed for PDAC may retain efficacy in selected tumors sharing common biological characteristics. Therefore, treatment selection based not only on anatomical origin but also on underlying biological features may represent a potential area for future investigation. From a biological perspective, dCCA is often classified as a pancreatobiliary-type tumor and shares substantial overlap with PDAC in terms of morphology, molecular characteristics, and key signaling pathways, including KRAS, TP53, and the TGF-β/SMAD axis. In addition, similarities in stromal reaction and tumor immune microenvironment have been reported (2, 28, 29, 40). These biological commonalities may, at least in part, influence treatment response patterns and provide a potential rationale for cross-tumor therapeutic approaches. Such biological overlap may, to some extent, influence response patterns to different chemotherapeutic regimens, thereby providing a potential theoretical rationale for cross-tumor therapeutic strategies. However, it should be emphasized that these hypotheses are primarily based on indirect evidence and require further validation in larger cohorts and prospective studies. In the present case, the absence of patient-specific genomic profiling limited further confirmation of these molecular similarities at the individual tumor level. Future investigations incorporating genomic sequencing may help clarify whether specific molecular alterations contribute to the observed therapeutic sensitivity and may further define biologically selected populations suitable for cross-tumor therapeutic strategies. In addition, the tumor in this case demonstrated a PD-L1 CPS of 30, suggesting a relatively active tumor immune microenvironment, which may, at least in part, be associated with the favorable response to combined immunotherapy and chemotherapy (41). Although the tumor demonstrated a relatively high PD-L1 CPS, PD-L1 expression alone cannot fully characterize the complexity of the tumor immune microenvironment. Comprehensive characterization of immune infiltrates, including CD8^+^ cytotoxic T cells, CD4^+^ helper T cells, FOXP3^+^ regulatory T cells, macrophage polarization (CD68/CD163), and tertiary lymphoid structures, may provide additional mechanistic insights into the favorable response observed in this patient. However, comprehensive immune microenvironment profiling was not performed because this retrospective single-case report was not initially designed as an immune biomarker-focused study. Future investigations integrating multidimensional immune profiling with clinicopathological features are warranted to better elucidate the biological basis of response to PD-1/CTLA-4 bispecific immunotherapy in distal cholangiocarcinoma. Beyond PD-L1 expression and immune cell composition, other molecular biomarkers, including mismatch repair (MMR) status, microsatellite instability (MSI), and tumor mutational burden (TMB), have been recognized as important factors associated with response to immune checkpoint inhibitors across various malignancies. These biomarkers may provide complementary information regarding tumor immunogenicity and may contribute to a more comprehensive understanding of immunotherapy responsiveness. However, MMR immunohistochemistry and MSI/TMB analyses were not performed in the present case because they were not included in the routine pathological and molecular evaluation during clinical management, and this retrospective single-case report was not initially designed as a biomarker-driven study. Future studies integrating molecular biomarkers with immune microenvironment profiling are needed to further clarify the determinants of response to PD-1/CTLA-4 bispecific immunotherapy in distal cholangiocarcinoma. Nevertheless, current evidence indicates that although PD-L1 expression in biliary tract cancers may show a potential predictive signal, its overall value remains controversial and insufficient as a standalone and reliable biomarker for treatment response. Meanwhile, immune checkpoint inhibitors combined with chemotherapy have demonstrated survival benefits in advanced biliary tract cancers (11, 42–44). However, in the context of CCA—particularly in the neoadjuvant or conversion setting for dCCA—available evidence remains limited (45, 46). Therefore, this case suggests that, in selected patients with borderline resectable CCA, dual immune checkpoint blockade combined with chemotherapy may have potential as a conversion strategy. However, the favorable therapeutic response observed in this patient should not be attributed solely to elevated PD-L1 expression, but rather to the combined influence of multiple factors, including tumor biology, immune microenvironment, chemosensitivity, and treatment-induced downstaging (47–49). From a surgical perspective, successful conversion therapy facilitated radical resection while avoiding arterial reconstruction, highlighting the potential role of neoadjuvant treatment in improving resectability and expanding surgical options for locally advanced pancreatobiliary malignancies (50, 51). In this case, the indication for surgery was primarily based on radiological response, improved resectability, and multidisciplinary evaluation rather than pathological response assessment. Although formal tumor regression grading (TRG) was not available, the resected specimen demonstrated successful local control that enabled R0 resection. Future studies investigating neoadjuvant or conversion therapy strategies for distal cholangiocarcinoma should incorporate standardized pathological response assessment, including TRG evaluation, to better characterize treatment efficacy. In addition, emerging circulating biomarkers such as ctDNA may provide dynamic information regarding treatment response and minimal residual disease. However, serial ctDNA monitoring was not performed in this case because this retrospective report was not designed as a biomarker-monitoring study. Future prospective investigations incorporating liquid biopsy approaches may further improve response assessment and risk stratification in distal cholangiocarcinoma undergoing conversion therapy. From a clinical perspective, this case highlights two important lessons. First, determination of the primary site in periampullary and pancreatobiliary tumors should not rely on a single diagnostic modality. Although EUS-FNA and immunohistochemistry are valuable for confirming malignancy and pancreatobiliary differentiation, accurate diagnosis requires integration of imaging findings, anatomical location, and histopathological features. Second, the biological overlap between PDAC and dCCA may provide a rationale for shared therapeutic strategies (13, 14). In the present case, AG chemotherapy combined with PD-1/CTLA-4 dual immune checkpoint blockade achieved a favorable response, suggesting that biologically informed treatment approaches may offer potential benefit in selected patients with borderline resectable or locally advanced pancreatobiliary malignancies. Nevertheless, further prospective studies are needed to validate this strategy.

In conclusion, this case not only highlights the diagnostic challenge of dCCA mimicking PDAC but also underscores the limitations of treatment strategies strictly based on organ origin in tumors of the pancreatobiliary junction. Under specific biological contexts, cross-tumor therapeutic approaches—such as combining non–tumor-specific chemotherapy regimens with PD-1/CTLA-4 dual immune checkpoint blockade—may hold potential clinical value and may improve resectability through neoadjuvant or conversion strategies. However, these findings are based on a single case observation and should be interpreted with caution. Further validation in larger cohorts and prospective studies is required to clarify their efficacy and to define the appropriate patient population, ultimately contributing to more precise and individualized treatment strategies for pancreatobiliary malignancies.

## Data Availability

The original contributions presented in the study are included in the article/supplementary material. Further inquiries can be directed to the corresponding authors.
